# Parents Rate Problematic Video Streaming in Adolescents: Conceptualization and External Assessment of a New Clinical Phenomenon Based on the ICD-11 Criteria of Gaming Disorder

**DOI:** 10.3390/jcm12031010

**Published:** 2023-01-28

**Authors:** Kerstin Paschke, Ann-Kathrin Napp, Rainer Thomasius

**Affiliations:** 1 German Center for Addiction Research in Childhood and Adolescence (DZSKJ), University Medical Center Hamburg-Eppendorf (UKE), Martinistrasse 52, D-20246 Hamburg, Germany; 2 Department of Child and Adolescent Psychiatry, Psychotherapy, and Psychosomatics, University Medical Center Hamburg-Eppendorf (UKE), Martinistrasse 52, D-20246 Hamburg, Germany

**Keywords:** streaming disorder, ICD-11, parental rating scale, binge watching, behavioral addiction

## Abstract

In recent years, video streaming (VS) increased substantially. Adolescents are at significant risk of presenting problematic VS patterns associated with a spectrum of mental-health difficulties. Because VS platforms rely on similar mechanisms as digital games, the ICD-11 criteria for Gaming Disorder (GD) have been successfully implemented to measure Streaming Disorder (StrD) in adolescents. For proper diagnoses, external rating scales are urgently required in addition to self-reports. The Streaming Disorder Scale for Parents (STREDIS-P) was created and validated in a representative sample of *n* = 891 adolescent-parent dyads. Mental health problems were assessed with standardized instruments. Confirmatory factor analysis was conducted to examine the underlying factor structure. Cutoff scores were determined using ROC analysis. Accordance between parental and adolescents’ self-ratings was calculated. Consistent with the results of previous validation studies for screening instruments assessing similar phenomena based on ICD-11-GD criteria, two factors, cognitive-behavioral symptoms and negative consequences, were confirmed. STREDIS-P demonstrated good to excellent internal consistency, criterion validity, and discriminatory power. Accordance with adolescents’ self-ratings was moderate. STREDIS-P is the first screening tool for assessing StrD in adolescents by parental ratings. It is highly relevant for conceptualizing a new phenomenon in clinical routine and academic research.

## 1. Introduction

### 1.1. Popularity of Video Streaming

Video streaming (VS), i.e., the on-demand or live retrieval of video content on professional or user-generated online platforms [1], has gained significant popularity during the last years in all age groups including infancy, childhood, and adolescence [2,3,4]. During the last decade, the number of subscribers to platforms like Netflix has exponentially increased [5]. Based on recent statistics, the platform YouTube counts over 2 billion monthly active users worldwide [6]. The COVID-19 pandemic has accelerated this development due to reduced social contacts, home schooling, and a lack of alternative leisure activities [7,8,9,10]. Currently, about half of German adolescents use VS platforms on a daily basis [2]. According to this large representative study, VS is the most popular and most frequently used digital activity in adolescents besides the use of social media (SM) platforms.

### 1.2. Video Streaming Versus Social Media Use

On multipurpose platforms like YouTube or TikTok, boundaries between VS and SM are more and more reduced [11,12]. However, VS is characterized by passive (i.e., pure consumption) in contrast to active (i.e., participating) SM use patterns with the latter involving active interactions like commenting on, discussing, liking, or sharing content [13].

### 1.3. Addictive Mechanisms of VS

Most research on digital-media-immanent factors increasing user bonding, and, thus, potentially fostering addictive use patterns, have focused on digital games [14,15]. However, parallels can be found on VS platforms. These include:

1. Service-design mechanisms: The availability of video content is almost unlimited and independent of temporal, local, or situational circumstances with intuitive user interface and no or low purchasing costs.
2. Emotional-design mechanisms: Microtargeting refers to the collection, analysis, and processing of user data to create user profiles for personalized offers and content suggestions tailored to individual preferences. A high quality of design and content built upon psychological mechanisms to attract attention and curiosity as well as the continuous presentation of new stimuli promote user bonding via behavioral and expectation-modulating processes. Experiences of immersion result on the one hand while due to unlimited content no natural end occurs on the other hand. Per default setting, the auto-play function on platforms will automatically start the next video. Unfinished content increases the probability of its consumption (Zeigarnik effect) [16].The regular observation that default settings are not adjusted due to human aspirations for minimal effort is known in business psychology as nudging, and is specifically considered in the design of user interface elements to influence the choice of behaviors in the digital environment [17].
3. Persuasive-design mechanisms: Personalized messages and content presentations make use of (intermittent) reinforcement-learning strategies (operant conditioning) to consolidate usage patterns and avoid extinction. The consolidation of behavioral automatisms and habits result. Psychosocial aspects are addressed by promoting social comparisons leading to the fear of missing out (FOMO) via the presentation of rankings and user feedbacks. Moreover, the scarcity and urgency of resources might be suggested by limited availabilities of offers to make use of human cognitive biases.

### 1.4. Problematic VS

Considering these underlying mechanisms and the high popularity of VS, it is surprising why there has been only little research attention on potential addictive effects of VS to date. Research on binge watching, i.e., viewing several episodes of a television series in one sequence [18], suggests the occurrence of addictive symptoms in relation to specific usage patterns, such as losing control, omitting alternative activities and obligations, feeling guilty, lying, showing withdrawal symptoms, and experiencing negative health-, social, and work-related, sequelae [19]. In this context, compared to regular binge-watching patterns, problematic ones are characterized by higher frequency and longer duration usage [20,21]. Based on studies with (young) adults, problematic patterns are linked to depressive and anxious symptoms as well as loneliness, psychological stress, and insomnia [22,23]. Yet, series binge watching resembles only one subentity of VS. A reduction of the conceptualization on it seems to be insufficient since it ignores the large number of alternative VS users. Moreover, the key characteristic of a user-controlled content retrieval applies to all VS offers [1].

### 1.5. Assessessment of Problematic VS

With a new research field, a valid and reliable conceptualization is needed to secure valid operationalization and comparability of results. To date, assessment has been mainly limited on motives, engagement, and basic addiction criteria in adults with binge-watching (cf. six core components of addiction model) [24]. It includes the Television Viewing Motivation Scale and Questionnaire of Excessive Binge-Watching Behaviors [25], Problematic Series Watching Scale [26], Series Watching Engagement Scale [27], Watching TV Series Motives Questionnaire and Binge-Watching Engagement and Symptoms Questionnaire [20], as well as the Binge-Watching Addiction Questionnaire [28]. With the inclusion of the digital-behavioral addiction Gaming Disorder (GD) into the latest version of the International Classification of Diseases (ICD-11) [29], standardized diagnostic criteria are now available that have been already successfully applied to related phenomena such as Social Media Use Disorder (SMUD) [30]. Adapted for disordered VS (i.e., streaming disorder, StrD), these describe a loss of control over VS, rising prioritization of VS over alternative activities, and the persistence or increase of VS despite negative sequelae over minimum 12 months. Importantly, the VS patterns must lead to clinically relevant distress or impairment of functioning in personal, social, educational, occupational, and economical areas of life [31]. Notably, due to their unspecificity, the frequency and duration of usage are no criteria of addictive patterns [23,26,28,32]. A structured and reproducible conceptualization is essential to separate potentially problematic from the majority of regular leisure users, and to avoid overpathologizing [33].

### 1.6. Involvement of Parents in Adolescent Assessment

Adolescents are especially at-risk to develop addictive behaviors as immature cognitive control mechanisms meet a fully developed neural reward system, which leads to increased sensitivity to motivational cues (cf. neurobiological imbalance model of adolescence) [34]. Depending on their age, developmental stage, and symptom severity, introspection might be limited. Therefore, in child and adolescent psychiatry settings it is common practice to involve guardians in the diagnostic process [35]. Parental ratings could be repeatedly shown to add valuable information, e.g., for the screening of problematic SM use [36]. However, despite the urgent need for research and clinical practice, no instrument to externally assess disordered VS has yet been available.

### 1.7. Aims of the Study

To close this significant gap and contribute to the conceptualization of a new phenomenon, the objectives of the current study included: (1) developing a parental screening instrument for StrD in adolescents (Streaming Disorder Scale for Parents, STREDIS-P); (2) characterizing its psychometric characteristics; (3) validating it in a representative sample of parents and their children (aged 10 to 17 years); (4) calculating the accordance between the ratings of parents and adolescents.

## 2. Materials and Methods

### 2.1. Participants and Procedure

In all, 1128 parents together with one respective child between 10 and 17 years (N_dyads_ = 2256) participated in a representative online survey. Data acquisition was realized by the German market and opinion polling company forsa, between 19 May and 6 June 2021. The selection of households was based on a random sampling procedure to ensure representativity regarding sex, age, and the region of residence. More details regarding the procedures can be found in Paschke, Napp et al. [31]. The study was authorized by The Local Psychological Ethics Commission at the Center for Psychosocial Medicine of the University Medical Center Hamburg-Eppendorf (LPEK-0307), carried out in compliance with the relevant national and institutional committees on human experimentation, and conformed with the Declaration of Helsinki. Together with their respective child, parents provided informed consent before participating in the study and could withdraw from the study at any point for any stated reason.

### 2.2. Measures

#### 2.2.1. VS Patterns

In the introduction to the questionnaire, participants received a definition of VS to clearly separate it from SM use. Accordingly, VS was referred to as the passive retrieval of online videos either from professional and/or user-generated VS platforms, i.e., users would only consume and not provide, share, comment on, or like any content. Platforms included on-demand versus live streaming services on the one hand and mono-versus multipurpose services on the other hand (e.g., Netflix, YouTube, Twitch, TikTok). Problematic VS patterns were measured by providing parents with STREDIS-P. STREDIS-P was created by clinicians and researchers experienced in the field of pediatric behavioral addictions. It was based on the ICD-11 criteria of GD as already successfully applied in psychometrically profound self- and parental questionnaires to assess GD [37,38] and SMUD [30,36]. Parents indicated their agreement with nine statements on a 5-point-Likert scale (strongly disagree (0)—strongly agree (4)) under consideration of the past 12 months with higher scores resembling more problems. The tenth item was related to problem, conflicts, or difficulties frequencies due to VS and answered by choosing one out of four response options (not at all (0) to nearly daily (3)). A score of ≥2 was considered significant regarding the ICD-11-time criterion. Items and corresponding ICD-11 criteria are shown in Table 1. Adolescents rated their VS patterns with the Streaming Disorder Scale for Adolescents (STREDIS-A) to investigate accordance between their and their parents’ answers. They fulfilled the criteria of StrD when the two subscale-sum scores (cognitive-behavioral symptoms and negative consequences) reached cut-offs (subscale 1 > 11 and subscale 2 > 6) and the time criterion was fulfilled [31]. Internal consistency of STREDIS-A was excellent for the study sample (Cronbach’s α = 0.91).

To date, no standardized questionnaire is available to assess adolescent problematic VS by parental ratings. Therefore, the parental-judgement adaptee of the Young Diagnostic Questionnaire (PYDQ) was administered [39]. PYDQ is an adaptation of the established YDQ by Young [40] which assesses problematic Internet use based on the criteria for pathological gambling of the fourth edition of the Diagnostic and Statistical Manual of Mental Disorders (DSM-IV); [41]. Notably, items 3, 5, and 6 of the PYDQ are related to ICD-11 GD criteria (Table 1). Parents were instructed to think of VS only when answering the eight items of the one-factorial polythetic scale with dichotomous answer type (yes (1) versus no (0)). Higher total scores were related to more problems. Originally, a cutoff ≥ 5 was applied and repeatedly used in a significant amount of studies [42]. Yet, more conservative approaches have been suggested for better differentiation [43]. Therefore, to avoid overestimation of problematic patterns, the condition that items 3 and/or 5 and item 6 were answered with yes was added to the standard cutoff. Internal consistency of PYDQ was acceptable in the current sample (α = 0.78).

VS frequency and duration were operationalized by asking parents about the typical number of usage days per week and usage time on week (school) days and weekend (leisure) days (in minutes). The latter two were summarized by calculating a mean value.

#### 2.2.2. Psychological Measures

Standardized scales on psychological stress perception, depressive and anxiety symptoms, loneliness, and insomnia were applied to measure construct validity. The criteria were chosen based on research on series binge watching [19,22]. Scales included: the 4-item version of the Perceived Stress Scale (PSS-4); [44], the 9-item Patient Health Questionnaire (PHQ-9) adapted for adolescents [45], the 2-item Generalized Anxiety Disorder Scale (GAD-2); [46,47], the 6-item Revised UCLA Loneliness Scale (R-ULS); [48,49,50] and the 7-item version of the Insomnia Severity Index (ISI); [51,52]. In all scales higher scores reflected more symptoms/ problems. Internal consistencies were good to acceptable in the study sample (PHQ-9: Cronbach’s α = 0.90; GAD-2: α = 0.81; R-ULS: α = 0.88; ISI: α = 0.70) with exception of PSS-4 (α = 0.59). Hence, PSS-4 was not considered for further analyses.

As a measure of scholastic performance, the final grades sum (past term) in German, Mathematics, and the first foreign language was calculated with individual grades ranging from very good (1) to failed (6) and, thus, lower scores meaning better performance.

### 2.3. Calculation

All calculations were carried out with the statistical program package R [53].

#### 2.3.1. Data Management

In all, 956 (84.75%) parents reported frequent VS of their children, i.e., VS at least once a week. These were considered for further analysis along with the corresponding child. 65 parent-child dyads had substantial missing values in STREDIS-A, -P, and PYDQ (missings > 1/3 of items) and were therefore abandoned. Thus, the final sample comprised 891 dyads (N_total_ = 1782). All non-substantial missing data were replaced by a multiple imputation procedure [54] using R package mice [55].

#### 2.3.2. Factor Analysis, Internal Consistency, and Criterion Validity

In all related ICD-11 questionnaires (GADIS-A/-P, SOMEDIS-A/-P, STREDIS-A) a two-dimensional factor structure had been previously shown (with factor 1 reflecting impending or manifest consequences and factor 2 cognitive-behavioral symptoms). To validate structure, confirmatory factor analysis (CFA) with robust minimal residuals (OLS) was applied to STREDIS-P. Model goodness of fit was based on five criteria: χ^2^/df ratio < 5, root mean square error of approximation (RMSEA) < 0.08, standardized root mean squared residual (SRMR) < 0.08, Tucker-Lewis Index (TLI) ≥ 0.95, comparative fit index (CFI) ≥ 0.95 [56]. Cronbach’s α and McDonald’s ω were estimated as measures of internal consistency [57].

In all related ICD-11 scales total scores resembled a reliable measure to assess criterion validity. Accordingly, correlation scores were computed between STREDIS-P sum score and VS days per week, mean VS time per day, total scores of PYDQ, PHQ-9, GAD-2, R-ULS, ISI, and the sum of school grades applying Spearman rank (VS days per week) and Pearson (all other variables) correlation tests [58,59].

#### 2.3.3. Sensitivity and Specificity

The R package pROC [60] was applied to perform receiver operating characteristic (ROC) curve analyses on STREDIS-P subscale sum scores. Sensitivity and specificity were determined and Youden’s criterion was employed to receive cutoffs for optimal prediction of StrD based on the PYDQ classification described above. 95% confidence intervals (CI) were calculated based on 999 bootstrapping replications. Goodness of differentiation between StrD and Non-StrD groups was resembled by the area under curve (AUC) value.

Adolescents with and without StrD were then compared regarding sex proportion by χ^2^ test and Cramer’s V for effect size estimation. Moreover, potential differences in age, VS days per week, mean VS time per day, SOMEDIS-A factor 1 and 2, PYDQ, PHQ-9, GAD-2, R-ULS, and ISI total scores as well as sum of school grades were evaluated by Multivariate Analysis of Variance (MANOVA) with post-hoc Scheffé tests and effect size Cohen’s d [58,61].

#### 2.3.4. Accordance

Accordance between ratings of parents and their respective child was investigated by applying Pearson correlations between the two factor sum scores of STREDIS-P and STREDIS-A. Additionally, StrD classifications retrieved from both questionnaires were compared within a 2 × 2-contingency table with absolute frequencies and relative agreement. The accordance rate between external and self-ratings was computed by (unweighted) Cohen’s к [62].

## 3. Results

### 3.1. Sample Description

Table 2 depicts relevant sample characteristics.

### 3.2. Factor Structure

CFA fit indices yielded mixed results regarding the underlying two-factor model: CFI of 0.995 and TLI of 0.994 were excellent and SRMR of 0.047 was acceptable. Yet, χ^2^/df ratio (χ^2^(26) = 239.77, *p* < 0.001, ratio = 9.22) and RMSEA value (RMSEA = 0.096, 95% CI (0.085, 0.107)) indicated poor fit [56]. However, data was significantly better modeled by two compared to one factor (χ_diff_^2^(1) = 88.988, *p* < 0.001; one-factor model fit indices: χ^2^/df ratio = 15.40 (χ^2^(27) = 415.98, *p* < 0.001); CFI = 0.992; TLI = 0.989; SRMR = 0.066; RMSEA = 0.127.

**Figure 1:**
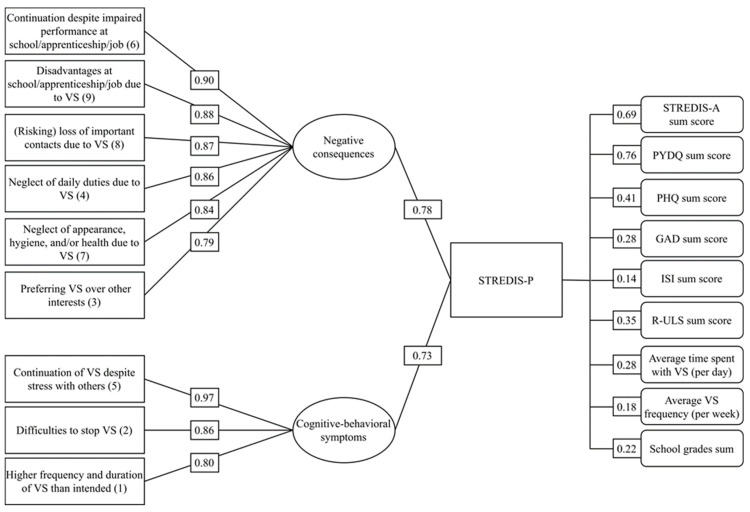
CFA factor loadings on the latent STREDIS-P factor 1 (negative consequences) and STREDIS-P factor 2 (cognitive-behavioral VS symptoms) are shown on the left. In the center the explained variance proportions of the two factors are depicted. Spearman (average VS frequency) and Pearson correlation coefficients (all other) of the STREDIS-P sum score with criteria are presented on the right. All factor loadings and correlations were significant with *p* values < 0.001. Abbreviations: STREDIS-P = Streaming Disorder Scale for Parents; STREDIS-A = Streaming Disorder Scale for Adolescents; PYDQ = Parental Young Diagnostic Questionnaire; PHQ = Patient Health Questionnaire; GAD = Generalized Anxiety Disorder Scale; ISI = Insomnia Severity Index; R-ULS = Revised UCLA Loneliness Scale; VS = video streaming.

On the one hand, highest loadings on factor 1 were observed by STREDIS-P items 3, 4, and 7 to 9. These reflect impending or manifest consequences due to VS. On the other hand, highest loadings on factor 1 occurred for items 1, 2, and 5, which mirror cognitive-behavioral symptoms of StrD (Figure 1). Yet, it must be noted that item 5 was not specifically representative for factor 2 since it showed also a substantial loading on factor 1. A significantly positive value between 0.79 to 0.97 was found for all standardized coefficients. Factors 1 and 2 significantly correlated by r = 0.65. Factor 1 explained a variance proportion of 0.78 and factor 2 of 0.73.

STREDIS-P items including the time criterion showed moderate to strong correlations (r = 0.40–0.73, Table 3). Relative item-response frequencies are presented in Table 4.

### 3.2.1. Internal Consistency

Excellent internal consistency of STREDIS-P was reflected by Cronbach’s α (α = 0.93) and McDonald’s ω (ω = 0.95). For each subscale Cronbach’s α of 0.91 and McDonald’s ω of 0.91 based on factor 1 as well as Cronbach’s α of 0.86 and McDonald’s ω of 0.87 based on factor 2 were calculated indicating good to excellent internal consistency.

### 3.2.2. Criterion Validity

The STREDIS-P sum score correlated with all criteria variables in a significant positive manner (Figure 1, right column). Strong correlation was observed with the PYDQ sum score indicating excellent criterion validity with a comparable measure. Moderate correlation was found with the PHQ-9 sum score as a measure of depression mirroring good criterion validity. Moreover, weak correlations were computed for the measures of anxiety (GAD-2), loneliness (R-ULS), and scholastic achievement (sum of school grades) as well as for the temporal use items VS days per week and mean daily VS time. A significant but negligible correlation was calculated with symptoms of insomnia (ISI).

### 3.2.3. Sensitivity and Specificity

ROC curve analyses on the two STREDIS-A subscales suggested the following optimal cutoffs based on Youden’s criterion: 9.5 (95% CI (7.5, 11.5)) for subscale 1 with a specificity of 85.22% (95% CI (75.57,91.42)), sensitivity of 90.38% (95% CI (80.67, 98.08)), an AUC value of 93.5% (95% CI (90.6, 96.4)), and an accuracy of 84.96%.; 6.5 (95% CI (6.5, 8.5)) for subscale 2 with a specificity of 80.45% (95% CI (76.75, 91.66)), sensitivity of 80.77% (95% CI (65.38, 92.31)), an AUC value of 87.7% (95% CI (82.8, 92.6)), and an accuracy of 79.80%. According to the AUC values, good to excellent differentiation can be assumed by both subscales.

When applying the cutoffs > 9 for subscale 1, > 6 for subscale 2 and under consideration of the ICD-11-time item (symptoms at least for longer periods or daily), 8.75% (95% CI [6.9, 10.61]) of the adolescents were classified with StrD (*n* = 78). When comparing adolescents based on their classification within a MANOVA, significant differences were found for all dependent variables except for age and the number of VS days per week (Pillai score (1732) = 0.33, F(11,72) = 32.00, *p* < 0.001). In more detail, strong differences were found regarding STREDIS-A subscale 1 and 2 sum scores, and PYDQ sum score with significant higher values in the StrD group. Moderate effects were observed for VS days per week, GAD-2 sum score, R-ULS sum score, and sum of school grades, again with higher values in the StrD group. Furthermore, ISI sum score was higher for adolescents with than without StrD with a small effect size. Among adolescents with StrD, the proportion of girls was significantly smaller (χ^2^ (1) = 16.65, *p* < 0.001) with a small effect size. Frequencies and statistical comparisons of adolescents with and without StrD are provided in Table 5.

### 3.2.4. Accordance

The total (r = 0.69, *p* < 0.001) scores as well as the factor 1 (r = 0.68, *p* < 0.001), and factor 2 scores (r = 0.60, *p* < 0.001) of STREDIS-P and STREDIS-A showed strong positive correlations. The time criterion scores between external and self-rating correlated moderately (r = 0.52, *p* < 0.001).

StrD classification of adolescents according to STREDIS-P and STREDIS-A was associated with Cohen’s к = 0.49 (95% CI (0.38, 0.60)) and a concordance of 93.5%. On a factorial level, Cohen’s к = 0.52 was calculated for subscale 1 and к = 0.45 for subscale 2. Thus, concordance between the ratings of parents and their respective children can be classified as moderate. For the time criterion, к = 0.55 was computed between both questionnaires also reflecting moderate concordance. Frequencies and accordance rates of adolescents screened with and without StrD by both judgments are shown in Table 6.

## 4. Discussion

Over the past decade, rising concern occurred regarding the addictive potential of intensive series streaming, i.e., binge watching [19]. However, besides watching TV series, VS platforms provide a far greater offer leading to high popularity and user numbers. Thus, a theory-based conceptualization of problematic VS is urgently needed to allow proper diagnosis and early intervention.

Adolescents are a risk group for developing disordered use patterns due to the popularity of VS platforms in this age group and ongoing brain maturation leading to higher vulnerability for digital addictions [63]. This risk seems to have increased under the COVID-19 pandemic with significant impact on adolescent mental wellbeing [64,65]. Parents are usually the first noticing potential problems in their children. Therefore, the aim of this study was to introduce a conceptualization based on the ICD-11 criteria of GD as the basis for a standardized parental assessment tool, STREDIS-P.

### 4.1. Psychometric Scale Properties

The 10-item STREDIS-P showed robust psychometric properties in a large representative parent-child sample with good to excellent internal consistency, criterion validity, and discriminatory power to differentiate adolescents with and without StrD, as well as moderate concordance between parental and adolescent ratings. The assumption of a two-factorial structure considering cognitive-behavioral symptoms and a clinical significance of these by negative consequences suggested by the innovated ICD-11 concept and the biaxial model of addiction, was supported by CFA [66,67]. Whereas one factor (factor 2) describes a VS pattern of increased frequency and duration and an inability to stop, the other factor (factor 1) refers to a loss of contacts, withdrawal, poor health, and reduced educational achievements as results of the VS pattern. If negative consequences have not yet been observed, a hazardous VS pattern can be assumed [29]. This structure is similar to conceptually related questionnaires on GD [37] and SMUD [30,68] as well as the self-rating scale on StrD [31]. Yet, items resembling the neglect of daily duties showed factor loadings that do not clearly support symptoms or consequences as an interaction might take place here. Moreover, the two factors were strongly correlated. Therefore, although the clinical significance of the two-factor approach is striking, from a psychometric point of view the use of the STREDIS-P total score in future multivariate analyses might be favored.

### 4.2. Problematic versus Unproblematic VS

A reliable differentiation between problematic and unproblematic usage patterns enables a better understanding of this new phenomenon, including etiology and interventional consequences [36]. The estimated cutoff for the subscale on cognitive-behavioral symptoms is identical to the self-rating scale. However, compared to the adolescent threshold, a lower parental threshold was identified for the subscale on negative consequences of the streaming behavior. This might reflect parents’ higher specificity and foresight for resulting problems. Given the high popularity of VS in adolescents and widespread intensive-use patterns, especially under the COVID-19 pandemic, target-group adapted thresholds are important to reduce false positive and false negative classifications of StrD [69].

Adolescents with StrD based on STREDIS-P classification scored higher on the parental version of YDQ to describe problematic VS based on DSM-IV criteria of pathological gambling, showed higher VS frequency and duration, more symptoms of loneliness, depression, anxiety, and insomnia, and lower school performance than adolescents without StrD.

According to the systematic review and meta-analysis of Marciano et al. [64], one aspect that might have fostered increased usage time in adolescents in general is FOMO, i.e., a state characterized by an anxiety of social exclusion based the feeling of difficulty to catch up with video content releases necessary for participation in social discussions. FOMO could have already been shown to be associated with problematic social media use [70,71]. Moreover, studies with adult problematic binge-watchers also reported higher VS intensities [20]. In line with our findings, an increased mental distress could be shown in studies on (problematic) binge watching [18,19,22,72,73,74], problematic SM and Internet use [75,76,77,78] as well as higher screen times in adolescents in general [79,80,81,82]. This might be associated with the usage motive to escape from reality and deal with loneliness [25,32,83,84]. Moreover, the presented data were collected during the COVID-19 pandemic. In this context, the above-mentioned systematic review discusses lower mood levels in relation to reduced physical activity under increased usage times [64].

In the recent systematic review of Alimoradi et al. [22] on binge watching among adults, greatest associations were found between binge watching and stress, as well as anxiety. Furthermore, the authors report associations with loneliness and insomnia, but also depression and sleep problems emphasized during the COVID-19 pandemic.

The group of positively screened adolescents included more boys than girls. This is in contrast to the finding on the self-rating-based classification where no sex difference was observed [31]. However, diverging results of potential sex differences are known from research on problematic SM use [36,85,86,87,88]. Furthermore, parents might be more sensitive to potentially problematic digital-media use in boys compared to girls based on different attributions [89].

### 4.3. Prevalence of Parent-Rated StrD

Applying the estimated cutoffs on parental ratings, 8.75% (95% CI (6.9, 10.61)) of the adolescents fulfilled the criteria for StrD. Parental rating of SMUD (5.93%; 95% CI (4.43, 7.44)) [68] showed similar prevalence although it was higher for GD (4.8%, 95% CI (3.3, 6.2)) [38]. Moreover, it is comparable to adolescent (prepandemic) prevalence estimations of anxiety (6.5%; 95% CI (4.7, 9.1)) and disruptive disorders (5.7%; 95% CI (4.0, 8.1)) but higher than those of depression (2.6%; 95% CI (1.7, 3.9)) and attention deficit hyperactivity disorder (3.4%; 95% CI (2.6, 4.5)) [90]. It must be noted that most of the latter prevalence rates were not only based on parental ratings and the COVID-19 pandemic had an impact on adolescent mental health in general with increased stress, anxiety, and depression [91,92]. However, the comparably high prevalence of StrD strengthens the need for more research on this new phenomenon.

### 4.4. Accordance between Parental and Adolescent Ratings

Keeping in mind the lower cutoff for subscale 1, the prevalence based on parental ratings is higher compared to adolescent ratings (4.69%, 95% CI (3.35, 6.03)). This finding is in line with a moderate concordance of external and self-rating. Moreover, on a descriptive level slightly higher prevalence rates were also found based on parental ratings on SMUD and GD [38,68]. Parents might be more critical and worried about their children’s behaviors or symptom severity on the one hand resulting in higher sensitivity for (negative consequences of) problematic behavior or potential exaggeration [89,93,94]. On the other hand, adolescents might tend to underestimate problems due to social desirability [95], age-related reduced introspective abilities [96], reduced functions of self-regulating and executive control functions associated with problematic use patterns [97], or symptom denial and concealment connected to addictive disorders [98].

Based on the review of De Los Reyes and Kazdin [93], discrepancies between parent-child judgements in the assessment of psychopathology are common with modest levels of agreement (ranging from 0.19 to 0.52) and can be explained by child, parent, family, and context characteristics. Usually, agreement is lower for adolescents compared to children and internalizing compared to externalizing disorders. According to the authors, no single measure or method exists that provides the gold standard in the diagnostic process. Hence, the combination of self- and external ratings shows the highest diagnostic value [35]. Together with the high prevalence and mental-health related issues, this underlines the potential significance of STREDIS-P to close an important gap for adequate conceptualization and valid screening instruments based on different perspectives. STREDIS-P can contribute to the active and standardized involvement of parents in the diagnostic process. In clinical routine, parents are usually the ones to initiate medical help for their children. Thus, their ratings allow a first evaluation of symptoms as an add-on to their children’s ratings or when the latter are not accessible due to reduced introspective abilities at a young age, symptom denial, or reluctance. Together with the diagnostic assessment by the experienced clinician, the detection of StrD will be improved by an early start of interventions for successful symptom reduction, and the prevention of severe consequences and chronification.

### 4.5. Strengths and Limitations

The strengths of this study include the highly up-to-date topic, the large representative parent-child sample, and the use of multiple standardized questionnaires to assess the validity and discriminatory potential of STREDIS-P. However, the following limitations should be noted: Firstly, highest concurrent validity would have been obtained by clinical face-to-face interviews. Usually, the benefit of full and uninfluenced answers is to the cost of sample size, justifying the online assessment of large data at this early stage of research. Secondly, missing data, a common problem of online surveys and with young-age samples, might have reduced representativity, but was accounted for by multiple imputations. Thirdly, future studies should include objective criteria as logged streaming times. Fourthly, no standardized instrument to evaluate StrD in adolescents was available as a comparative measure of STREDIS-P. Therefore, PYDQ was used for comparison since it has been largely applied in adolescent-parent samples and has large similarities with the DSM-5 criteria of Internet gaming disorder [99]. Fifthly, the data was acquired during the COVID-19 pandemic. This might have influenced usage times, mental distress, and the prevalence rates of StrD. Different measurement points and a prospective design would be desirable for future studies to add information on re-test reliability and potential cause-and-effect relationships. Furthermore, investigations on the influence of different VS patterns and service structures might be highly interesting.

## 5. Conclusions

The high popularity of VS services and increasing usage times in adolescents demand conceptualization and reliable differentiation between unproblematic and problematic VS patterns. In child and adolescent psychiatry, standardized parental reports are regularly used to supplement or, in the case of unavailability, substitute self-reports for sophisticated diagnosis. The newly developed 10-item screening instrument, STREDIS-P, is based on the ICD-11 criteria of GD. It is easy and economical to apply and could prove good to excellent internal consistency, reliability, and criterion validity in the assessment of StrD in adolescents by parental ratings. Thus, STREDIS-P closes an important gap for the detection of StrD in scientific and clinical contexts to promote comparable research, initiate proper treatment, and prevent chronification.

## Figures and Tables

**Table 1:** STREDIS-P items with corresponding ICD-11 criteria and PYDQ items.

ICD-11 Criteria	STREDIS-P Items
(Corresponding PYDQ Items)	Thinking of the last 12 months, how strongly do you agree with the following statements?
A. Impaired control over streaming (e.g., onset, frequency, intensity, duration, termination, context).	My child often uses video-streaming more frequently and longer than he/she planned to or agreed upon with me or my partner. ^a^
(Has your child repeatedly made unsuccessful efforts to control, cut back, or stop Internet use? Does your child stay online longer than originally intended?)	2. My child often cannot stop using video-streaming even though it would be sensible to do so or, for example, I have told him/her to stop. ^a^
B. Increasing priority given to streaming to the extent that streaming takes precedence over other life interests and daily activities.	3. My child often does not pursue interests outside the digital world, because he/she prefers to use video-streaming services. For example, he/she does not meet with friends/partner in real life, does not attend sports clubs/societies, does not read books, or make music because of using video-streaming services. ^a^
	4. My child neglects daily duties, because he/she prefers video-streaming. Daily duties include, e.g., doing grocery shopping, cleaning, tidying up after himself/herself, tidying up his/her room, fulfilling obligations for school/apprenticeship/job. ^a^
C. Continuation or escalation of streaming despite the occurrence of negative consequences.	5. My child continues using video-streaming services even though it causes him/her stress with others. This means, e.g., stress with me or my partner, siblings, friends, partner or teachers because of video-streaming. ^a^
(Has your child jeopardized or risked the loss of a significant relationship, job, educational, or career opportunity because of the Internet?)	6. My child continues using video-streaming services although it harms his/her performance at school (or apprenticeship/job). For example, he/she is late, does not participate in class, neglects homework and gets worse grades because of video-streaming. ^a^
D. The behavior pattern is of sufficient severity to result in significant impairment in personal, family, social, educational, occupational, or other important areas of functioning.	7. Due to using video-streaming services, my child neglects his/her appearance, personal hygiene, and/or health. For instance, he/she sleeps less, eats unhealthily, and/or exercises less because of video-streaming. ^a^
	8. Due to using video-streaming services, my child risks losing important contacts or has lost them already. This includes contacts with partners, friends, acquaintances, or family. ^a^
	9. Due to using video-streaming services, my child has disadvantages at school/apprenticeship/job. For example, he/she got bad (final) grades, is unable to continue to next grade or does not graduate, he/she has no place for training or studying, and/or got a poor reference or a warning/ dismissal as a result of video-streaming. ^a^
E. The pattern of streaming behavior may be continuous or episodic and recurrent and normally evident over a period of at least 12 months.	10. In the past year, how often did your child experience the conflicts or difficulties described in the statements 1 to 9 due to using video-streaming services? Did this only occur on single days, during longer periods of several weeks to months, or was it almost daily? ^b^

Notes: STREDIS-P = Streaming Disorder Scale for Parents; PYDQ = Parental Young Diagnostic Questionnaire; ICD-11 = 11th revision of the International Classification of Diseases. ^a^ response options: 5-point Likert-Scale: “strongly disagree”—“strongly agree”; ^b^ response options: “not at all”, “only on single days”, “during longer periods”, “almost daily”.

**Table 2:** Sociodemographic characteristics of final sample ^a^.

Variables/Categories	Adolescents N [% (95% CI)]/Mean (SD; Range)	Parents N [% (95% CI)]/Mean (SD; Range)
Absolute frequency	959	959
**Sex**		
Male	472 [52.97 (49.70–56.25)]	435 [48.82 (45.54–52.10)]
Female	419 [47.03 (43.75–50.30)]	456 [51.18 (47.90–54.46)]
Age in years	13.51 (2.16; 10–17)	45.64 (7.36; 28–72)
**Parent-child relationship**		
Biological child	820 [92.03 (90.25–93.81)]	
Adoptive child	4 [0.45 (0.01–0.89)]	
Stepchild	44 [4.94 (3.52–6.36)]	
Other ^b^	23 [2.58 (1.54–3.62)]	
**Education level ^c,d^**		
High	463 [55.99 (52.60–59.37)]	213 [23.93 (18.32–29.55)]
Medium	300 [36.28 (33.00–39.55)]	598 [67.19 (59.53–74.85)]
Low	64 [7.74 (4.54–10.93)]	65 [7.30 (5.39–9.22)]
**Occupation ^e^**		
Full time employment/school attendance	843 [95.25 (93.85–96.66)]	542 [60.58 (57.45–63.63)]
Part time employment/apprenticeship	30 [3.39 (2.20–4.58)]	264 [30.14(27.32–33.11)]
Other ^f^	12 [1.36 (−0.32–3.03)]	85 [9.54 (4.22–14.86)]
**Place of residence**		
Urban living ^g^	167 [18.74 (16.18–21.31)] 724 [81.26 (78.69–83.82)]	
Rural living		
**Psychological measures**		
GAD-2 sum score ^h^	0.92 (1.32; 0–6)	
ISI sum score ^i^	8.57 (6.48; 0–28)	
PHQ-9 sum score	4.74 (4.96; 0–27)	
R-ULS sum score ^j^	11.47 (4.37; 6–24)	
PYDQ sum score		2.13 (2.06; 0–8)

Notes: N = absolute frequency; CI = confidence interval; SD = standard deviation; GAD = Generalized Anxiety Disorder Scale; ISI = Insomnia Severity Index; PHQ = Patient Health Questionnaire; PYDQ = Parental Young Diagnostic Questionnaire; R-ULS = Revised UCLA Loneliness Scale. ^a^ dyads with frequently streaming using adolescents, i.e., adolescents use streaming at least once a week; ^b^ not specified; ^c^ for parents: highest level achieved—high = bachelor/master’s degree to doctorate (Ph.D); medium = secondary school leaving certificate (Realschulabschluss)/ university entry qualification (Abitur)/completed apprenticeship; low = no or lower school-leaving certificate (Hauptschulabschluss); for adolescents: (prospective) school leaving certificate (based on the current school performance)—high = university entry qualification (Abitur), medium = secondary school certificate (Realschulabschluss), low = no/special-school (Förderschulabschluss)/ lower school certificate (Hauptschulabschluss); ^d^ no response adolescents *n* = 64, no response parents *n* = 1, not specified parents *n* = 14; ^e^ Item not presented to adolescents younger than 14 years, no response adolescents *n* = 6; ^f^ for adolescents: university students, in voluntary service, military service, other occupation, or unemployed; for parents: unemployed, job seeking, welfare recipient, pensioners, disabled, trainee, student, no specification; ^g^ areas with ≥5000 residents; ^h^ no response adolescents *n* = 29; ^i^ no response adolescents *n* = 9; ^j^ no response adolescents *n* = 6.

**Table 3:** Inter-item Pearson correlation of STREDIS-P items ^a^.

Items ^b^	1	2	3	4	5	6	7	8	9	Time Criterion
1	1.00									
2	0.72	1.00								
3	0.46	0.54	1.00							
4	0.55	0.60	0.60	1.00						
5	0.63	0.70	0.60	0.73	1.00					
6	0.49	0.56	0.54	0.68	0.71	1.00				
7	0.40	0.48	0.58	0.61	0.60	0.63	1.00			
8	0.42	0.49	0.63	0.56	0.63	0.65	0.68	1.00		
9	0.43	0.48	0.52	0.56	0.62	0.75	0.63	0.71	1.00	
Time criterion	0.62	0.63	0.55	0.61	0.62	0.61	0.55	0.56	0.53	1.00

Notes: STREDIS-P = Streaming Disorder Scale for Parents. ^a^ based on total sample of *n* = 891 parents; ^b^ for the description of items, refer to Table 1. The items of factor 2 are highlighted in gray.

**Table 4:** Relative item-response frequency of STREDIS-P items (in %) ^a^.

STREDIS-P Items ^B^	Response Options				
	Strongly Disagree	Somewhat Disagree	Partially Agree/Partially Disagree	Somewhat Agree	Strongly Agree
Item 1	17.28	24.47	32.32	20.20	5.72
Item 2	23.91	30.08	22.90	16.72	6.40
Item 3	40.18	31.65	17.51	7.52	3.14
Item 4	28.62	28.28	25.36	13.24	4.49
Item 5	37.37	30.19	18.63	10.33	3.48
Item 6	50.39	26.37	12.12	8.31	2.81
Item 7	56.79	24.35	11.11	5.50	2.24
Item 8	52.75	29.85	10.66	4.71	2.02
Item 9	58.24	26.94	8.98	3.82	1.80
	not at all	only on single days	for longer periods	nearly daily	
Time criterion	30.75	55.33	11.45	2.47	

Notes: STREDIS-P = Streaming Disorder Scale for Parents. ^a^ based on the total sample of *n* = 891 parents; ^B^ for the description of items, refer to Table 1. The items of factor 2 are highlighted in gray.

**Table 5:** Frequencies and statistical comparisons of adolescents with and without StrD.

Variables Frequency [95% CI] Mean (SE)	No StrD	StrD	F-Value (MANOVA)	χ²/Post-Hoc Scheffé Tests	Effect Size ^a^
absolute frequency	813	78	-	-	-
relative frequency	91.25 [89.39; 93.1]	8.75 [6.9; 10.61]	-	-	-
female sex in %	49.2 [45.76; 52.64]	24.36 [14.83; 33.88]	-	16.65 ***	0.14
age	13.5 (0.08)	13.51 (0.21)	0.01 NS (*p* = 0.97)	-	0.01
STREDIS-P subscale 1 sum score	4.34 (0.14)	15.76 (0.44)	-	11.41 ***	2.84
STREDIS-P subscale 2 sum score	3.86 (0.09)	9.6 (0.19)	-	5.74 ***	2.19
STREDIS-P time criterion score	0.73 (0.02)	2.22 (0.05)	-	1.49 ***	2.63
STREDIS-A subscale 1 sum score	3.86 (0.09)	10.78 (0.44)	181.29 ***	6.8 ***	1.6
STREDIS-A subscale 2 sum score	4.37 (0.09)	7.9 (0.19)	116.68 ***	3.53 ***	1.22
PYDQ sum score	1.79 (0.06)	5.64 (0.21)	293.56 ***	3.85 ***	2.2
average number of VS days per week	5.45 (0.07)	5.74 (0.22)	2.75 NS (*p* = 0.10)	-	0.15
average VS time per day [in minutes] ^b^	197.01 (14.65)	285.41 (18.35)	29.91 ***	88.4 ***	0.67
PHQ-9 sum score	4.31 (0.16)	9.31 (0.68)	60.37 ***	5.0 ***	1.05
GAD-2 sum score	0.84 (0.04)	1.76 (0.2)	26.0 ***	0.92 ***	0.71
R-ULS sum score	11.19 (0.15)	14.51 (0.54)	43.15 ***	3.32 ***	0.78
ISI sum score	8.37 (0.22)	10.81 (0.79)	3.41 ***		0.38
grades sum ^c,d^	6.35 (0.27)	7.62 (0.37)	13.27 ***	1.27 ***	0.52

Notes: StrD = Streaming Disorder; MANOVA = Multivariate Analysis of Variance; χ² = chi-square; CI = confidence interval; NS = not significant; SE = standard error of means; VS = video streaming; STREDIS-A = Streaming Disorder Scale for adolescents; subscale 1 = negative consequences; subscale 2 = cognitive-behavioral symptoms; PYDQ = Parental Young Diagnostic Questionnaire; PHQ = Patient Health Questionnaire; GAD = Generalized Anxiety Disorder Scale; R-ULS = Revised UCLA Loneliness Scale; ISI = Insomnia Severity Index. ^a^ based on Cramer’s V for female sex and Cohen’s d for all other variables; ^b^ Mean of VS per week (school) day and weekend (leisure) day [in minutes]; ^c^ Sum of school grades in mathematics, German, and first foreign language (each ranging 1–6, with higher scores indicating worse performance); ^d^ during past school term. *** *p* ≤ 0.001.

**Table 6:** Absolute frequencies and accordance of adolescents positively and negatively screened with StrD.

	STREDIS-P +	STREDIS-P −	Accordance (%)
STREDIS-A +	32	12	72.73
STREDIS -A −	46	801	94.57
Accordance (%)	41.03	98.52	93.49

Notes: + positively screened, − negatively screened. Abbreviations: StrD = Streaming Disorder; STREDIS-P = Streaming Disorder Scale for Parents; STREDIS-A = Streaming Disorder Scale for Adolescents.

## Data Availability

The data presented in this study are available on reasonable request from the corresponding author (KP) after all results of the parent-child survey have been published.
